# Supplementary material for: Early-Enrichment Hit Discovery via Reversible-Work c(t) Estimation in Metadynamics (CTMD)
Source: bioRxiv. 2026 Feb 8:2026.02.05.703972. Preprint. [Version 1] doi: 10.64898/2026.02.05.703972 (PMC12889651; doi:10.64898/2026.02.05.703972)
Supplement: 1 [file NIHPP2026.02.05.703972v1-supplement-1.pdf]

bioRxiv preprint doi: https://doi.org/10.64898/2026.02.05.703972; this version posted February 8, 2026. The copyright holder for this preprint (which was not certified by peer review) is the author/funder, who has granted bioRxiv a license to display the preprint in perpetuity. It is made available under aCC-BY 4.0 International license.

Supplementary Information

Methods

Ligand selection

Each of the test systems has a very differing number of hits and decoys available to be tested. In order to standardize our experiment, we targeted a standardized hit rate of 10% where was a reasonable level to expecting from preliminary docking. We sampled as many true binders and decoys as possible ensuring a tanimoto similarity[24] of less than 0.4 between all ligands. This usually limited most systems to less than 50 molecules.

System selection and preparation

We hand-picked systems involving different classes of proteins from the Large-Scale Docking (LSD) database[25] and Schrodinger's public FEP data for JAK2 Kinase,[19] since these databases could provide initial guesses for the protein-ligand interaction pose as would be expected from most docking screens. The proteins selected here are CB1 and Alpha2A receptors (G-protein coupled receptors), JAK2 (a kinase) and AmpC (an enzyme).

The initial protein structure was retrieved from these databases and was prepared in UCSF Chimera[26] via Dock Prep, without changing the protonation state of any residue. This structure was saved in the default Protein Data Bank (PDB) format. For membrane proteins, CHARMM-GUI[27] was used to prepare the initial system, exporting the final structure with AMBER parameters. The ligands were parameterized using AMBER's GAFF2 forcefield[28] and all components were then assembled using the leap program provided in AmberTools.[29] Finally, Na^+ and Cl^- ions were added, and the system was neutralized with the ionic concentration set to 150 mM.

Molecular Dynamics

All simulations were performed on PMEMD patched with PLUMED version 2.9.2[30–32] with a 2 fs time step. Simulations were performed utilizing the AMBER ff19SB force field[33] for protein, TIP3P for water molecules[34] and, General Amber ForceField (GAFF2)[28] for all ligands. Temperature and pressure were kept at 303 K and 1 bar using the velocity rescale thermostat[35] and Parrinello-Rahman barostat.[36] The non-bonded interactions were calculated with a 10 Å cutoff, and long-range electrostatics were calculated using the particle-mesh Ewald (PME) method.[37]

Each system was first subjected to a short energy minimization for 15000 steps followed by heating it from 0K to 100K at a rate of 20K/ps and then from 100K to 303K at a rate of 2K/ps. The systems were then allowed to equilibirate for 1 ns at these temperatures with restraints (k=5 kcal/mol/Å^2) applied to all backbone atoms and with the same restrains applied only to C_α atoms for another 1 ns.

For the first stage of filtering, all ligands were subjected to an unbiased MD for 5ns. Any ligands that left the active site i.e. had a pose with RMSD higher than the CTMD cutoff (described later) were automatically given a score of 0. For the ligands that remained, multiple metadynamics trajectories were fired from the final frame of the 5ns MD. The collective variable (CV) biased was protein-aligned ligand RMSD where the protein atoms were aligned and the RMSD of the ligand atoms was calculated to the reference. The reference was taken to be the start of the unbiased MD instead of the end to honour the poses predicted by docking and the heavily penalize pose drift during unbiased MD. The final system sizes are provided in Table **CTMD Simulation Summary**.

bioRxiv preprint doi: https://doi.org/10.64898/2026.02.05.703972; this version posted February 8, 2026. The copyright holder for this preprint (which was not certified by peer review) is the author/funder, who has granted bioRxiv a license to display the preprint in perpetuity. It is made available under aCC-BY 4.0 International license.

CTMD Protocol

All Metadynamics simulations, unless explicitly mentioned used only this RMSD as the Collective Variable (CV) with a gaussian σ of 0.015 kJ/mol and a height of 1.75 kJ/mol, a bias factor of 10, and a pace of 1 ps and a run length of 5 ns.

Using RMSD as the CV allows biasing the ligand's coordinates relative to the protein in a completely unsupervised manner. This ensures that our method translates between systems with minimal fine-tuning of variables - which is extremely effective for drug discovery where it is difficult to know apriori the parameters that will provide enrichment without access to known binders.

$c(t)$ as computed in Eq. 1 serves as an excellent proxy for the total bias added, and represents the total work done through the external biasing.[14,15] We expect this value to remain reasonably stable for a given system. The simulation is terminated if the ligand RMSD ever crosses 6Å and stays above for 200ps. Keeping in line with the general approach of using minimum work to quantify free energy differences,[14] we repeat the unbinding metadynamics simulation 3 times, for a total of under 15 ns of biased MD, and take the lowest C(t) value. In this work, in order to achieve better statistics, we repeated the simulations 10 times, and in order to mimic a real-world scenario, all results reported are bootstrapped by repeatedly picking 3 random trajectories from these 10, and use the mean value from 250 such repeats. The error bars are a result of this process, and where applicable, subsampling of the true binders.

CTMD Simulation Summary

The following table summarizes the the number of binders and initial system size (for MD) picked from their respective databases in each study. Many targets had far more ligands available. However, whenever possible, binders and non-binders selected were filtered to have Tanimoto similarity below 0.4 amongst themselves. Where more than 5 distinct binders were available, only 5 binders were picked randomly for mutliple iterations of bootstrapping, and this number is used in calculating the base hit-rate.

System	Type	# of Ligands	# of Binders	# of Decoys	Base hit-rate	System Size
**JAK-2**	Soluble (Kinase)	41 ligands	14 binders	27 decoys	15.6%	41000 atoms
**AmpC**	Soluble(Enzyme)	28 ligands	4 binders	24 decoys	14.2%	42500 atoms
**CB1R**	Membrane Protein (GPCR)	28 ligands	4 binders	24 decoys	14.2%	75000 atoms
**A2AR**	Membrane Protein (GPCR)	33 ligands	6 binders	27 decoys	15.6%	69000 atoms

Other measures of enrichment

BEDROC is a normalized version of the Robust Initial Enhancement (RIE)[38] score where RIE weights *early* enrichment higher than the overall enrichment, which is captured by a metric like AUROC (Area Under ROC). It is defined as follows:

$$BEDROC(\alpha) := \frac{RIE(\alpha) - RIE_{min}(\alpha)}{RIE_{max}(\alpha) - RIE_{min}(\alpha)} \text{ with } RIE(\alpha) \sim \sum_{i=1}^{n} e^{-\alpha r_i}$$

Higher values of α encourage earlier enrichment. It is usually plotted for different values of α. The literature value of $\alpha = 20$ is commonly used to reduce the metric to one number[39] (reported here)

bioRxiv preprint doi: https://doi.org/10.64898/2026.02.05.703972; this version posted February 8, 2026. The copyright holder for this preprint (which was not certified by peer review) is the author/funder, who has granted bioRxiv a license to display the preprint in perpetuity. It is made available under aCC-BY 4.0 International license.

CTMD always *improves* hit rate compared to raw docking scores

The following plot compares BEDROC profiles using just DOCK 3.7 scores for all systems (except JAK2 which uses GLIDE XP scores) compared to using CTMD ranking. In all cases, CTMD clearly improves hit-rate (independent of the value of α chosen as weighting factor)

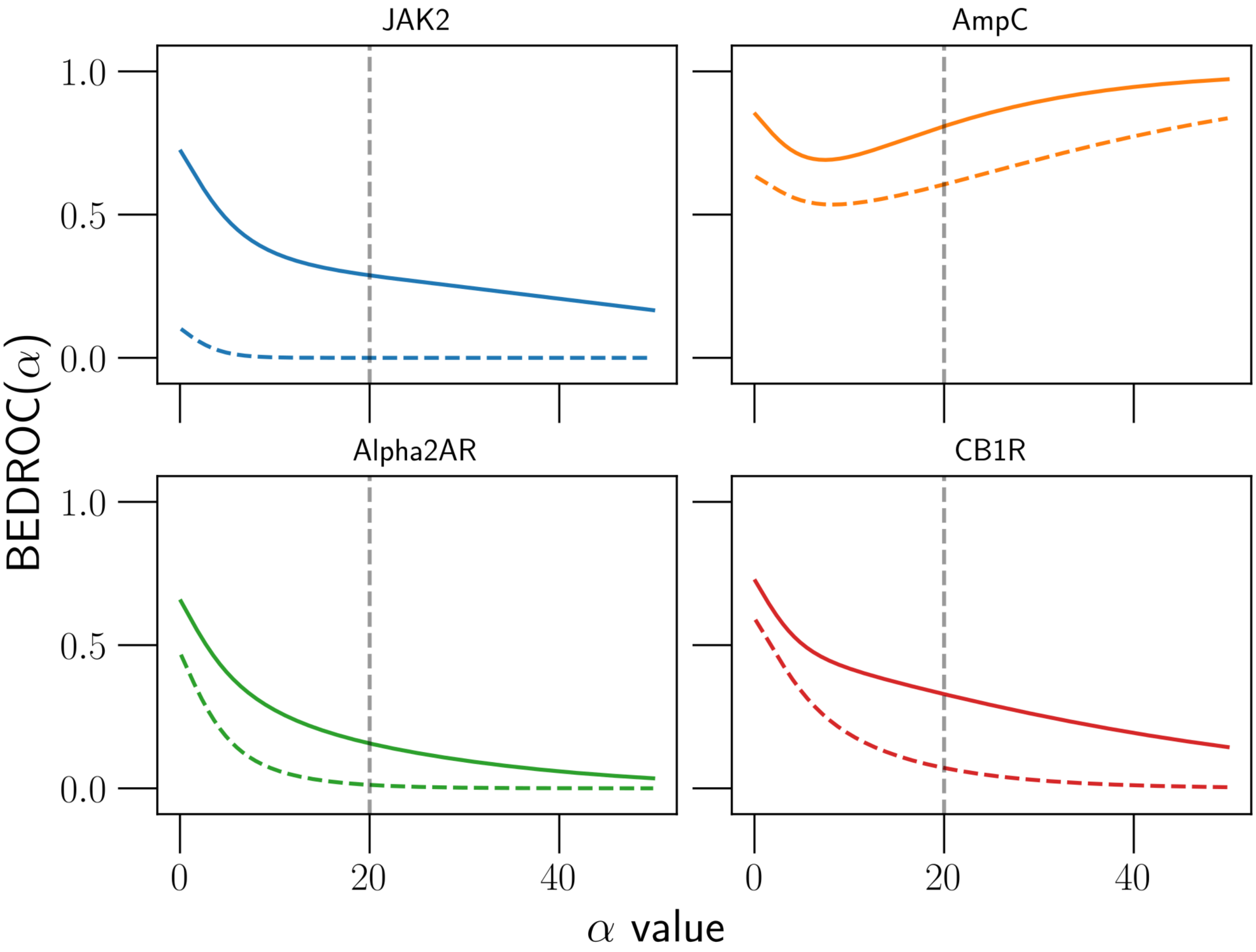

Figure 4: Plot of BEDROC profile as a function of α for each of the 4 systems tried. The solid line represents CTMD. The dashed line represents the docking score.

bioRxiv preprint doi: https://doi.org/10.64898/2026.02.05.703972; this version posted February 8, 2026. The copyright holder for this preprint (which was not certified by peer review) is the author/funder, who has granted bioRxiv a license to display the preprint in perpetuity. It is made available under aCC-BY 4.0 International license.

Training set similarity of binders in our benchmarks

Many of our benchmarks have ligands which are present (including affinity scores) in the training set for Boltz-2. Needless to say, Boltz-2 performs really well on these systems. Similarity to ligands obtained from CheMBL for each of the four targets with reported affinity (IC50, EC50, Kd, or Ki) values was assumed to be reasonable information for Boltz-2 to extrapolate "hits" from. Maxmimum common substructure (MCS) fraction was used as the metric for similarity, computed using RDKit's rdFMCS.[40]

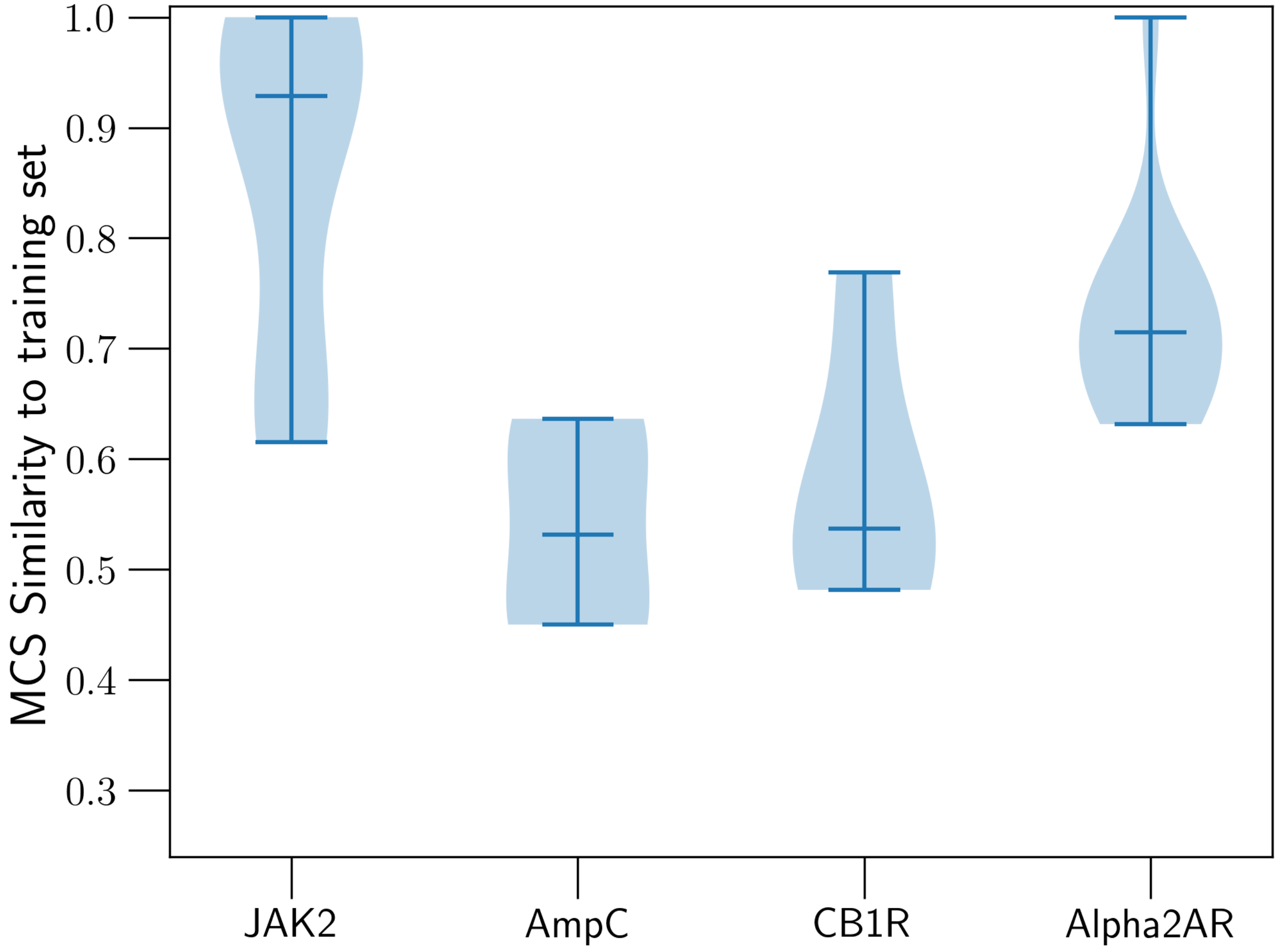

Figure 5: Distribution of Maxmimum common substructure (MCS) similarity of binders in our benchmark set to Boltz-2's training data. The horizontal line inside each distribution represents the median value.

bioRxiv preprint doi: https://doi.org/10.64898/2026.02.05.703972; this version posted February 8, 2026. The copyright holder for this preprint (which was not certified by peer review) is the author/funder, who has granted bioRxiv a license to display the preprint in perpetuity. It is made available under aCC-BY 4.0 International license.

Boltz-2 enrichment occasionally outperforms CTMD, but often due to memorization

The following plot compares BEDROC profiles comparing to using CTMD ranking with Boltz-2 affinity prediction. Boltz-2 is noticably unreliable, performing really well on systems with poor representation in the training data.

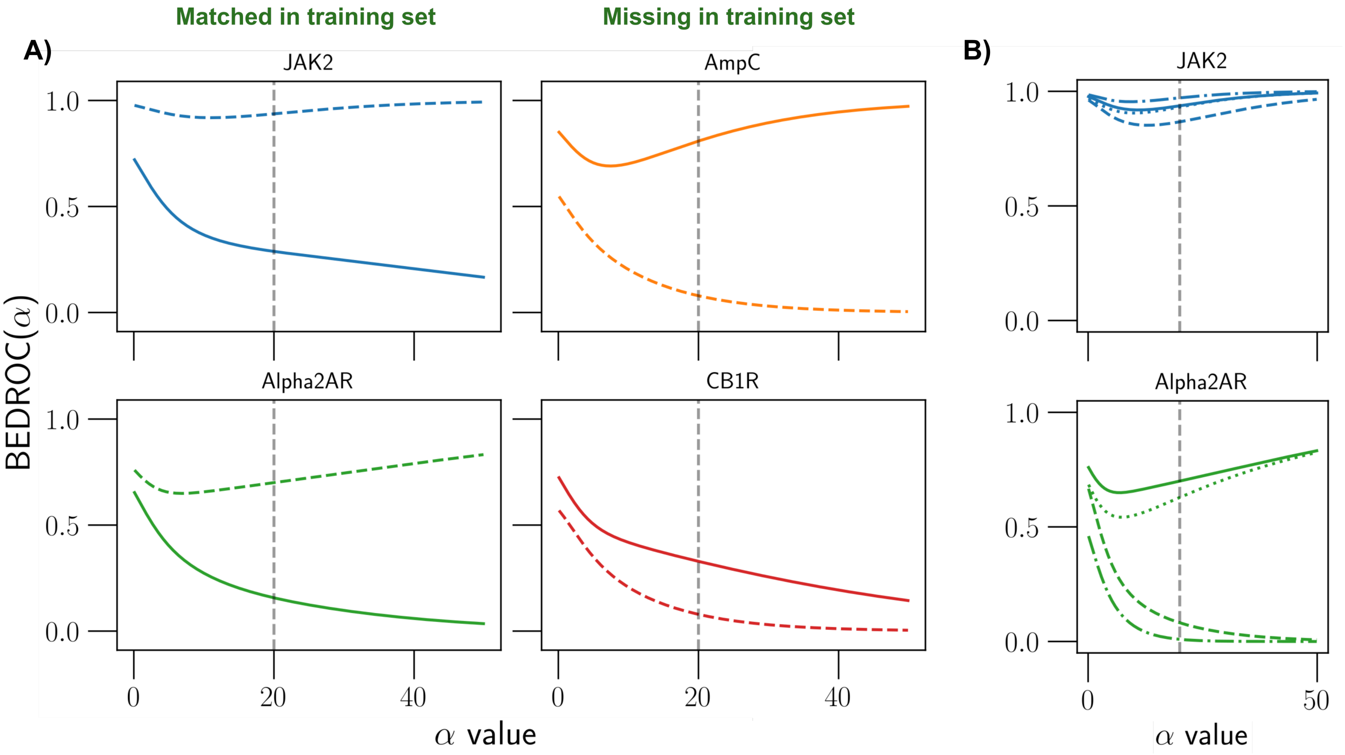

Figure 6: A) Plot of BEDROC profile as a function of α for each of the 4 systems tried. The solid line represents CTMD. The dashed line represents the Boltz-2. Notice that significant performance improvements are seen when ligands have high similarity to training set. B) Evidence of memorization in Boltz-2. Notice the early enrichment even when the active site is modified i) dashed - All to PHE; ii) dotted - All to ALA; iii) dot-dash - Flipped Polarity. The solid line is for the wild type

bioRxiv preprint doi: https://doi.org/10.64898/2026.02.05.703972; this version posted February 8, 2026. The copyright holder for this preprint (which was not certified by peer review) is the author/funder, who has granted bioRxiv a license to display the preprint in perpetuity. It is made available under aCC-BY 4.0 International license.

Evidence that mutations destroy the active site - DOCK 6 example

We have shown evidence of Boltz-2 memorization through examples where mutations to the protein that destroy the active site. Despite each mutant - especially ones to Phe and ones where polarity are flipped - showing a distinct drop in docking scores, Boltz-2 shows enrichment above random even with these pockets, indicating a high likelihood of memorization.

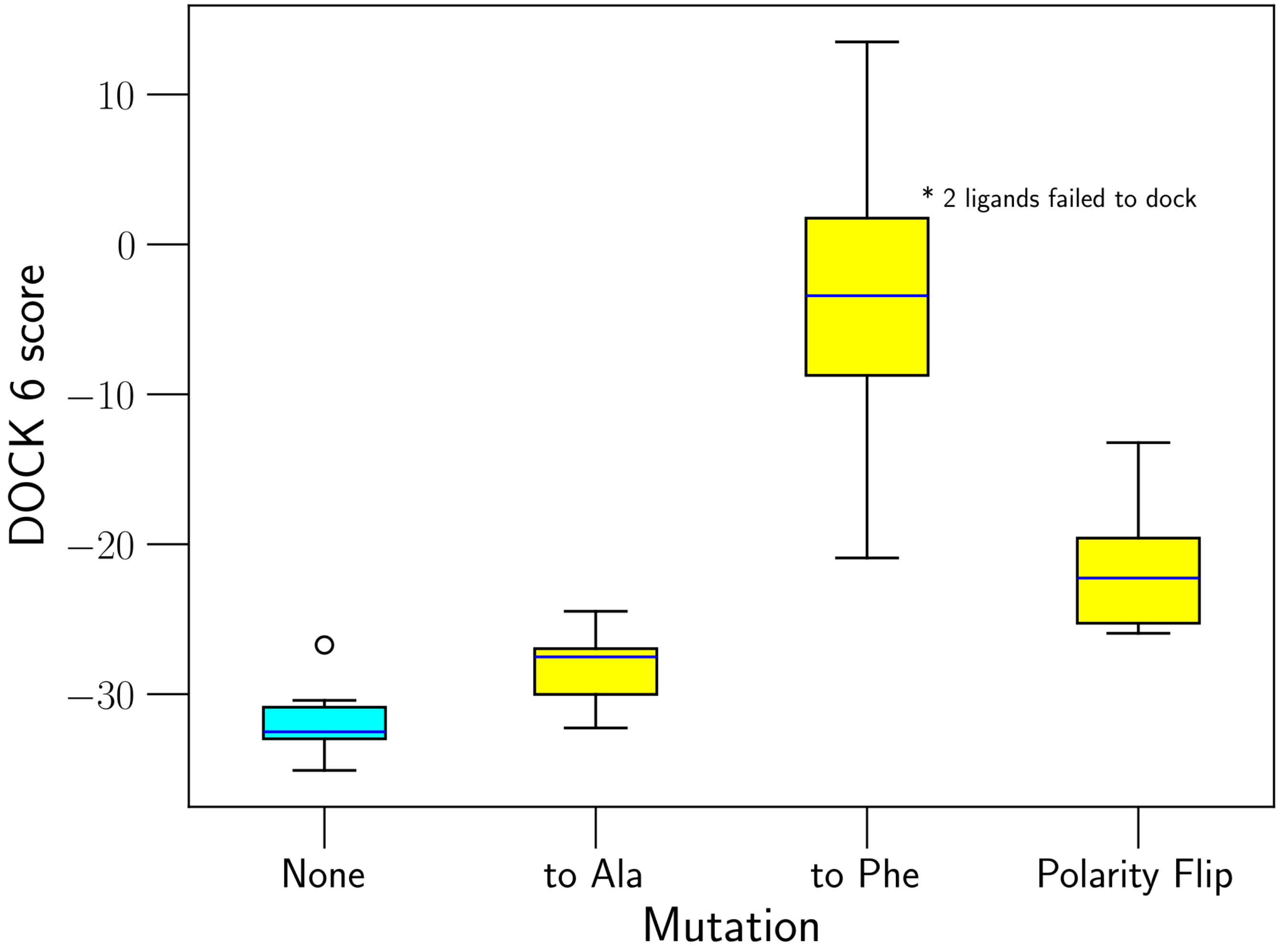

Figure 7: Increase in DOCK 6 score distributions when mutations to the active site are introduced. The blue horizontal lines in each box represent the medians of the distribution. The wild-type (no mutation) is shown in cyan. The mutants are shown in yellow.
